# Editorial: Experimental biology and medicine: new frontiers

**DOI:** 10.3389/ebm.2024.10069

**Published:** 2024-01-30

**Authors:** Steven R. Goodman

**Affiliations:** Department of Pediatrics, College of Medicine, University of Tennessee Health Science Center, Memphis, TN, United States

**Keywords:** experimental biology, medicine, frontiers, editorial, publishing partnership

*Experimental Biology and Medicine* (EBM) has transferred to the Gold open access publisher Frontiers with this being the seminal issue of the partnership. Authors can submit manuscripts through the Frontiers portal (https://www.ebm-journal.org/submission/submit).

The Society of Experimental Biology and Medicine (SEBM) was created in 1903 in the Northeast of the United States and its journal, then called the *Proceedings of the Society of Experimental Biology and Medicine*, was first published in June 1904. The *Proceedings of the Society of Experimental Biology and Medicine* was renamed *Experimental Biology and Medicine (EBM)* in 2001. I was honored to be selected as the Editor-in-Chief of EBM in 2006, with my first issue published in July of that year. When being interviewed for the position of Editor-in-Chief, I stated two goals: 1) to expand the number of categories from the seven that existed in 2006 by adding cutting edge interdisciplinary and multidisciplinary new categories and 2) to globalize the journal and, in so doing, globalize the Society. We have accomplished both goals over the last 17 years.

EBM now has twenty-two categories to which authors can submit manuscripts that encompass the breadth of modern biomedical research. Our categories cover the entire translational spectrum including basic, preclinical, clinical, clinical trial and population research. EBM’s focus is on biomedical research that is relevant to the practice of medicine. We publish original research articles, Brief Communications, Reviews, Mini Reviews, and at the Editor-in-Chief’s discretion Commentaries and Editorials.

In terms of global expansion EBM/SEBM now has offices and Global Editors on the six habitable continents. In addition to its US offices in Memphis, Tennessee, College Station, Texas, and Washington DC; EBM/SEBM now has offices in Taiwan (Tainan), the United Kingdom (London), China (Chengdu), Brazil (Campinas), Ghana (Accra), and Australia (Perth). The current 180 members of the Editorial Board are outstanding scientists coming from the global research community. With the accomplishments of broadening the scope and coverage of our categories and expanding to all habitable continents, EBM submissions have grown by over 6-fold since my becoming Editor-in-Chief, with the correlated growth of the impact of our content.

EBM is unique in its broad range of topical areas and its coverage of the complete translational research spectrum. I believe that building upon its strong history and current direction, EBM will become the premier journal that publishes biomedical research that has a direct impact on the practice of modern medicine. The new EBM-Frontiers partnership is committed to a fully open access model which makes our publications rapidly accessible to all researchers internationally. Advances in biomedical research require open, transparent, rapid communication between all researchers. It is this international collegial interaction which will have the greatest impact upon improving the health and wellbeing of all people around the globe.

I personally invite you to submit your work to *Experimental Biology and Medicine.* We will oversee your submission in the manner that we want our own work to be reviewed and judged. Together we will help transform global health through biomedical research.

